# Transport Properties of One-Step Compression Molded Epoxy Nanocomposite Foams

**DOI:** 10.3390/polym11050756

**Published:** 2019-04-30

**Authors:** Mario Martin-Gallego, Emil Lopez-Hernandez, Javier Pinto, Miguel A. Rodriguez-Perez, Miguel A. Lopez-Manchado, Raquel Verdejo

**Affiliations:** 1 Instituto de Ciencia y Tecnología de Polímeros, ICTP-CSIC, C/ Juan de la Cierva, 3, 28006 Madrid, Spain; m.martingallego@ictp.csic.es (M.M.-G.); emil.lopez@ictp.csic.es (E.L.-H.); lmanchado@ictp.csic.es (M.A.L.-M.); 2 Cellular Materials Laboratory (CellMat), Condensed Matter Physics Department, Faculty of Science, University of Valladolid, Campus Miguel Delibes, Paseo de Belén, 7, 47011 Valladolid, Spain; jpinto@fmc.uva.es (J.P.); marrod@fmc.uva.es (M.A.R.-P.)

**Keywords:** epoxy, foams, expandable microspheres, graphene, nanotubes, conductivity, syntactic foams

## Abstract

Owing to their high strength and stiffness, thermal and environmental stability, lower shrinkage, and water resistance, epoxy resins have been the preferred matrix for the development of syntactic foams using hollow glass microspheres. Although these foams are exploited in multiple applications, one of their issues is the possibility of breakage of the glass hollow microspheres during processing. Here, we present a straightforward and single-step foaming protocol using expandable polymeric microspheres based on the well-established compression molding process. We demonstrate the viability of the protocol producing two sets of nanocomposite foams filled with carbon-based nanoparticles with improved transport properties.

## 1. Introduction

Syntactic foams are a type of cellular material composed of pre-formed hollow glass microspheres, also called microballoons, that are bound together with a matrix [1]. These foams have largely been prepared using thermoset matrices, particularly epoxy resins (ER), where the hollow microspheres can easily be accommodated [2]. The outstanding properties of epoxy resins combined with the lightness of the microspheres are exploited in multiple applications, ranging from packaging materials for expensive components to core material in sandwich structures and deep-sea submersibles [3]. However, among their different issues is the possibility of breakage of the glass hollow microspheres during mixing and handling. 

An alternative to produce epoxy foams is the use of polymeric expandable microspheres (EMSs) as foaming agents since they provide better control over the pore morphology [2,4,5]. These microspheres are made of a thermoplastic polymer shell, around 3–7 μm thick, encapsulating a blowing agent, usually a saturated hydrocarbon with low boiling point. When heated, the polymeric shell gradually softens, and the liquid hydrocarbon begins to gasify and expand when the heat is removed, the shell stiffens and the microsphere remains in its expanded form. Kim and Kim [4] reported the use of these EMSs as a toughening method concluding it outperformed the usual hollow microspheres. Meanwhile, Wang et al. [5] proposed a two-step procedure, precuring and foaming, to produce an epoxy foam reinforced with glass fibers, montmorillonite and silica with these EMSs. They report differences in the cellular structure as a function of the precuring extent and foaming temperature, with a homogeneous distribution of the cells at a high precuring extent and foaming temperature, and small cell size at a high precuring extent and low foaming temperature. 

The advent of nanofillers has brought about a strategy to improve different polymer properties at low loading fractions [6] and has provided unique opportunities for the reinforcement of fine structures, such as fibers, thin films and foams. In particular, the use of carbon nanoparticles (CNPs) in foams has proven not only to increase the mechanical properties, but also to ease processability of high-performance thermoplastic foams [7] or to impart new functionalities, such as self-extinguishing grade [8], electrical conductivities, or EMI shielding, among others (see References [9,10] for reviews in the subject). The use of CNPs in epoxy foams has mostly been studied in syntactic foams with glass microballoons, whose concentration varies from 20 wt.% to 60 wt.% respect to the resin. Carbon nanotubes (CNTs) reinforced epoxy foams showed an improvement in compressive modulus by 35%–41% while the strength remained unchanged [11]. Guzman et al. [12] used functionalized CNTs and various commercially available microballoons to produce syntactic epoxy foams showing a significant increase in compressive strength and apparent shear strength. Carbon nanofibers and graphene platelets have also improved the mechanical properties of syntactic foams in tension mode [13,14,15] while the compressive modulus and strength were slightly affected [13,15,16,17]. The use of CNP has also been explored with epoxy foamed via EMSs. Bao and co-workers have developed a foaming protocol based on a two-step process followed by a traditional post-cure step of epoxy resins [18,19,20,21,22,23]. They have reported lower electrical percolation threshold and higher conductivity than those of the solid counterpart [18], and improvements in both electromagnetic interference shielding and sound absorption properties compared to unfilled samples [20,21,22]. 

Here, we present a straightforward and single-step process using expandable microspheres based on the well-established compression molding foaming. We demonstrate the viability of the process producing two sets of nanocomposite foams filled with carbon-based nanoparticles with improved transport properties.

## 2. Materials and Methods 

### 2.1. Materials

Diglycidyl ether of bisphenol-A epoxy resin (product number: 405493), and diethylene triamine curing agent (D93856) were purchased from Sigma-Aldrich (Darmstadt, Germany). Microspheres of Expancel (AkzoNobel 930 DU 120) were used as a foaming agent. These spheres consist of a thin thermoplastic shell filled with a hydrocarbon. According to the material datasheet of the manufacturer, the used EMSs are composed of an acrylonitrile/methacrylonitrile/methyl methacrylate copolymer, CAS number 38742-70-0, and two hydrocarbons, 2,2,4-trimethylpentane and isobutane. The average diameter of the unexpanded microspheres is around 30 μm and increases to a maximum of about 120 μm. Manufacturer information stated an expanding temperature between 122 °C and 132 °C and a maximum temperature of use between 191 °C and 204 °C. 

### 2.2. Synthesis of Carbon Nanoparticles

Aligned multi-walled carbon nanotubes (MWCNTs) were synthesized in-house by a chemical vapor deposition technique. Briefly, a mixture of 3 wt.% ferrocene in toluene was injected at a rate of 5 mL/h into a hot furnace (760 °C) using argon as carrier gas; the nanotubes grew on the inner wall of a quartz tube and present an oxygen content below 0.7 at. %. Thermally reduced graphene oxide (TRGO) was also synthesized in-house by the rapid thermal exfoliation/reduction of dried graphite oxide (GO) at 1000 °C under an inert atmosphere. GO was synthesized from natural graphite obtained from Sigma-Aldrich (universal grade, purum powder ≤ 0.1 mm, 200 mesh, 99.9995%) according to the Brödie method, which has previously been described by the authors [24]. Briefly, natural graphite and fuming nitric acid were added to a reaction flask and cooled to 0 °C. KClO_3_ was gradually added and the mixture was stirred for 21 h, maintaining the reaction temperature at 0 °C. Then, the mixture was diluted in distilled water and filtered until neutral pH. 

### 2.3. Preparation of ER Foams

First, the nanoparticles were dispersed using a three-roll calender device, EXAKT 80 E (EXAKT 80E, EXAKT Technologies, Inc. Oklahoma City, OK, USA), following the three-step protocol described in Table 1.

Then, the epoxy/nanofiller dispersion was degassed under vacuum until complete removal of the occluded air. Epoxy/nanoparticle, the stoichiometric amount of hardener and 7 wt.% of Expancel with respect to the resin were mechanically stirred at low rpm until a homogeneous blend was achieved. Then, the mixture was poured inside a square metallic mold (10 × 10 × 1.5 cm^3^) and placed in a hot press at 100 °C and 60 bars for 3 min and, subsequently, cooled down to room temperature maintaining the pressure. Three different EMS concentrations, 7 wt.%, 15 wt.% and 30 wt.% were initially considered based on the concentrations reported in syntactic foams. However, both 15 wt.% and 30 wt.% did not provide further density reductions compare to 7 wt.% and were discarded from the study. Foams were produced with two different densities varying the amount poured into the mold, low-density (LD) around 250 kg/m^3^ and high-density (HD) around 800 kg/m^3^. Finally, the foam was post-cured at 130 °C for 90 min. The foams were mechanized and 2 mm of solid skin was cut out from each side of the foam. The nanofiller concentrations were selected according to the viscosity of the dispersion. The maximum concentrations were 0.5 wt.% for MWCNTs and 1.5 wt.% for TRGO. 

### 2.4. Characterization

The density of a cubic sample was measured as its weight divided by its volume according to ASTM D 1622-03. The results were the average of at least three measurements.

The expansion of Expancel was analyzed using differential scanning calorimetry (DSC), Mettler Toledo 822e DSC. Measurements were carried out from room temperature up to 200 °C at a heating rate of 10 °C/min.

The morphology of the foams was determined by scanning electron microscopy (SEM, FEI SA, Hillsboro, OR, USA). The images were taken with a Philips XL30 ESEM at 25 kV. The samples were metallized with a 5 nm coating of gold/palladium. The morphology of the CNPs was observed by scanning (SEM) and transmission electron microscopy (TEM). TEM images were obtained on a Philips Tecnai 20 TEM apparatus (Field Electron and Ion Company, Hillsboro, OR, USA) using a voltage of 200 kV. SEM analysis was performed on a piece extracted from the walls of the quartz tube without coating. Meanwhile, the TEM samples were prepared by immersion of the TEM grid in a dilute solution of nanoparticles in THF and letting the solvent evaporate.

Thermal conductivity was measured by the transient plane source (TPS) technique using a thermal conductivity analyzer model HDMD (Hot Disk, Göteborg, Sweden). TPS is a standard technique that measures time-dependent energy dissipation of a sample [25]. It uses a thin disk that acts both as a temperature sensor and heat source and which is located between two samples of similar characteristics (area and thickness).

The electrical conductivity of the foam nanocomposites was determined on an ALPHA high-resolution dielectric analyzer (Novocontrol Technologies GmbH, Hundsangen, Germany) over a frequency range window of 10^−1^–10^7^ Hz at room temperature. The foams were held in the dielectric cell between two parallel gold-plated electrodes. The amplitude of the applied AC electric signal to the samples was 1 V.

## 3. Results and Discussion

Previous works by the authors have already analyzed the effect of the carbon nanoparticles used in this study in the rheology [26], curing kinetics [27], and transport properties, both thermal and electrical, of similar thermally cured epoxy resins [26,28,29]. These studies together with the authors’ knowledge on the viscosity hindrance of foaming in reactive systems [24,30,31,32,33] enable fixing the foaming processing temperature and time and the maximum concentration of nanoparticles mentioned above, respectively. 

**Figure 1:**
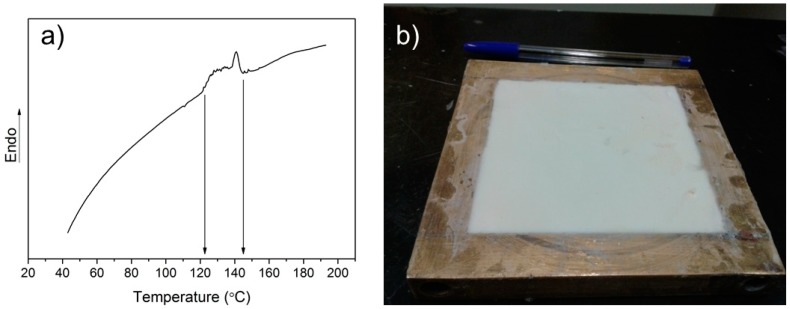
(**a**) Dynamic DSC thermogram of the expandable microspheres. (**b**) Fully expanded epoxy foam (100 °C and 3 min).

Several studies by Bao and coworkers [18,19,20,21,22,23] on epoxy/Expancel systems reported two-step foaming processes, composed of pre-curing at low temperatures (around 45 °C) and foaming (75 to 100 °C temperature range), followed by traditional epoxy post-curing. The authors stated their intention to adopt a single-step process but they encountered thermal degradation of the matrix. Here, we decided to optimize the one-step process based on a well-established industrially scalable foaming process, i.e., compression molding. To establish the appropriate foaming temperature, Expancel microspheres were initially characterized by dynamic DSC (Figure 1). The thermogram revealed the expansion process of the microspheres in the temperature range from 122 °C to 145 °C. 

Hence, an initial foaming trial was done at 125 °C. However, the exothermic curing reaction of the epoxy resin increased the temperature resulting in the eventual degradation of the sample after only 1 min. Hence, considering the previously studied cure kinetics [27], we decided to lower the temperature to 100 °C. At this temperature, the cure kinetics had been analyzed through rheology and MQ NMR showing a gel time around 2.7 min and a vitrification time around 4 min, respectively. Therefore, we fixed the compression molding within this time frame; obtaining the best results at 3 min (Figure 1b). Once the foaming process is completed, it is key to reduce the temperature of the mold below the Tg of the EMSs to fix the cellular structure, and also to avoid degradation of the matrix. The optimization of the foaming process for different hardeners and epoxy monomers should be done considering the reaction kinetics of the cure.

### 3.1. Morphology

**Figure 2:**
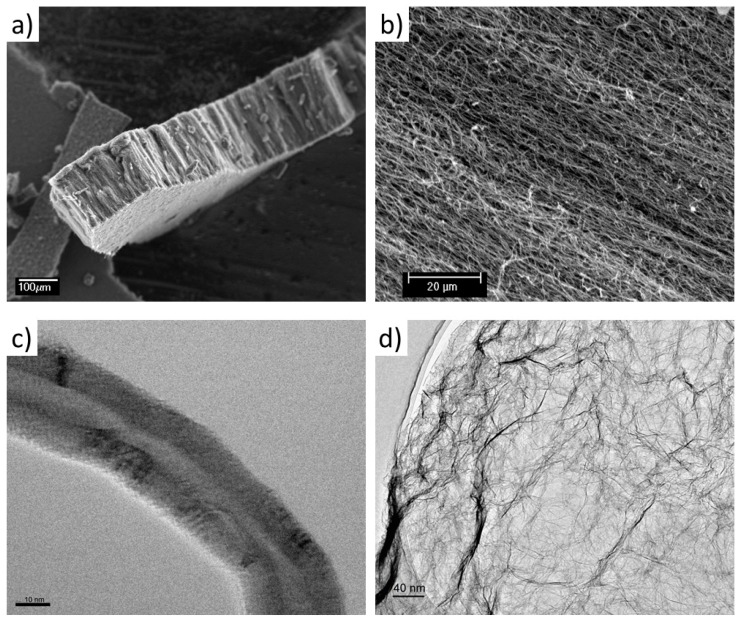
Morphology of in-house synthesized nanoparticles: (**a**) and (**b**) SEM and (**c**) TEM images of MWCNTs, (**d**) TEM image of TRGO.

The morphology of the nanofillers is presented in Figure 2. MWCNTs are aligned and disentangled; this characteristic would facilitate their dispersion in the epoxy resin. Their length, measured from the low magnification image, is approximately 160 µm, with an average diameter of 43.8 ± 12.7 nm, which results in an aspect ratio above 3600. Meanwhile, the morphology of the TRGO shows the characteristic wrinkled structure of the particle due to the thermal exfoliation and reduction to which it has been subjected. Full characterization of the TRGO and MWCNT used in this work is described elsewhere [29,34,35].

**Figure 3:**
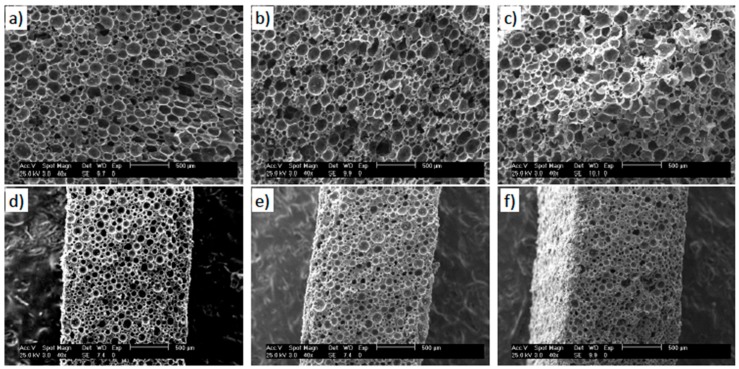
SEM images of the foams. (**a**) Neat ER-LD, (**b**) 0.5 wt.% MWCNT-LD, (**c**) 1.5 wt.% TRGO-LD, (**d**) neat ER-HD, (**e**) 0.5 wt.% MWCNT-HD, and (**f**) 1.5 wt.% TRGO-HD.

**Figure 4:**
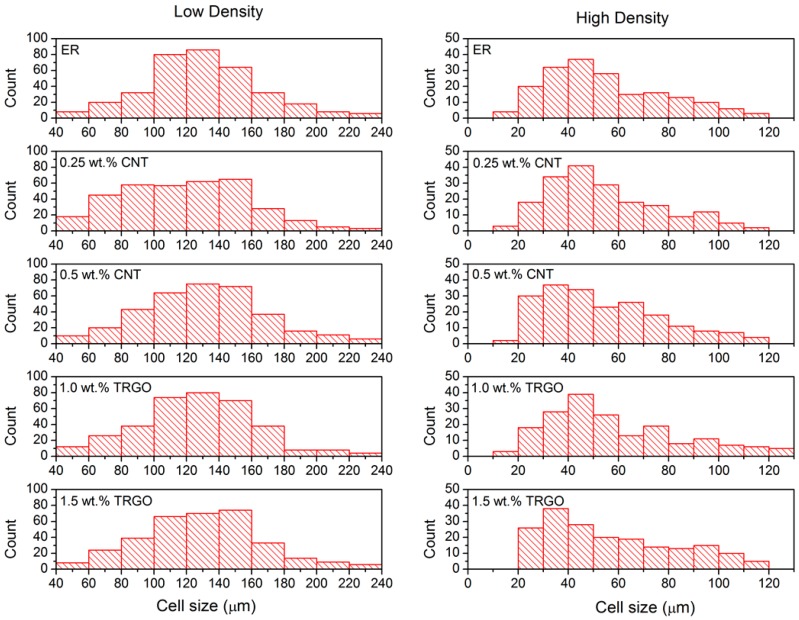
Cell size distribution of the foams.

The developed foams with the one-step process show a closed-cell, homogeneous and isotropic structure (Figure 3). Such closed cellular morphology is the result of the foaming agent, as the shell of the microspheres will be part of the cell wall. Foams within the same density set present fairly similar cell morphology and cell size distribution (Figure 4), with average cell size and wall thickness ranging from 125 ± 5 µm and about 1–2 µm to 56 ± 2 µm and 10–12 µm for low- and high-density foams, respectively (Table 2). The cellular morphology of previous studies presents either a bimodal cell size distribution or the presence of extremely large cells [5,18,20,21,22,23], neither of these two effects were observed in our samples (Figure 4). We ascribe this homogeneity of the cellular structure to the single-step foaming process and to the degassing of the CNP mixture.

**Figure 5:**
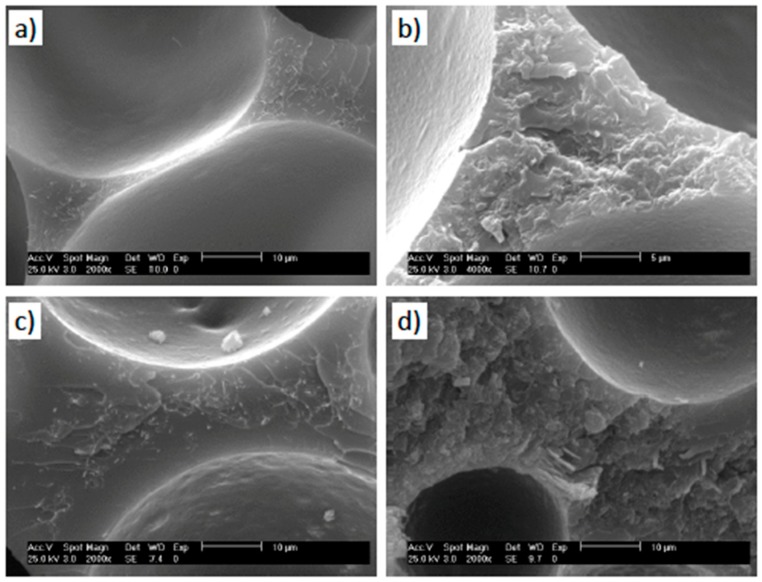
High magnification SEM images of the foams. (**a**) 0.5 wt.% MWCNT-LD, (**b**) 1.5 wt.% TRGO-LD, (**c**) 0.5 wt.% MWCNT-HD, and (**d**) 1.5 wt.% TRGO-HD.

Nanoparticles have been reported to act as nucleating points in different nanocomposite foams [9]. Here, we do not observe such an effect since foaming occurs through the expansion of the microspheres and we removed any occluded air. Individual MWCNTs and TRGO were visibly protruding from the polymer matrix and were uniformly distributed within both the struts and walls with no obvious aggregation (Figure 5).

Considering the initial value of the microspheres’ diameter before the expansion (30 µm) and the value of the cell size, we obtain an expansion factor of approximately 4.3 and 1.5 for low- and high-density foams, respectively. These expansion factors are related and agree well with the density reduction, from 1100 kg/m^3^ to around 250 kg/m^3^ and 850 kg/m^3^ for LD and HD, respectively (Table 2). LD expansion ratio is slightly higher than those obtained by other authors using EMSs in epoxy resins [5] which suggest an optimal level of expansion.

### 3.2. Thermal Conductivity

The cellular structure of polymer foams gives them very low thermal conductivity, which is a sought after property for insulating applications. However, some specific applications can require the transport of heat while maintaining lightweight characteristics. For example, improved thermal conductivity (λ) in polymer foams can be useful to eliminate temperature gradients and maintain a uniform temperature in the thermal insulation of the space shuttle [3], in mobile phones, or any electronic circuit. Therefore, the incorporation of nanofillers with superior thermal conductivity has been studied as a way to increase the thermal conductivity of foams [24,36]. 

Heat transfer can occur through three main mechanisms: conduction, convection, and radiation. However, convection is considered to be negligible in foams with cell size below 4 mm [2], while radiation has a minor contribution in systems with relative densities above 0.2 [37]. Since the developed foams satisfy these two requisites, the dominant heat transfer mechanism would be via conduction in both the solid and gas phases, which depends on the volume fraction of each phase, the cellular structure and the thermal conductivity of the solid phase. Furthermore, as we mentioned above, the cellular morphology of the foams is very similar among the samples with the same density. Therefore, the differences in the thermal conductivity of the nanocomposite foams with the same density should be ascribed to the presence of the nanofillers.

**Figure 6:**
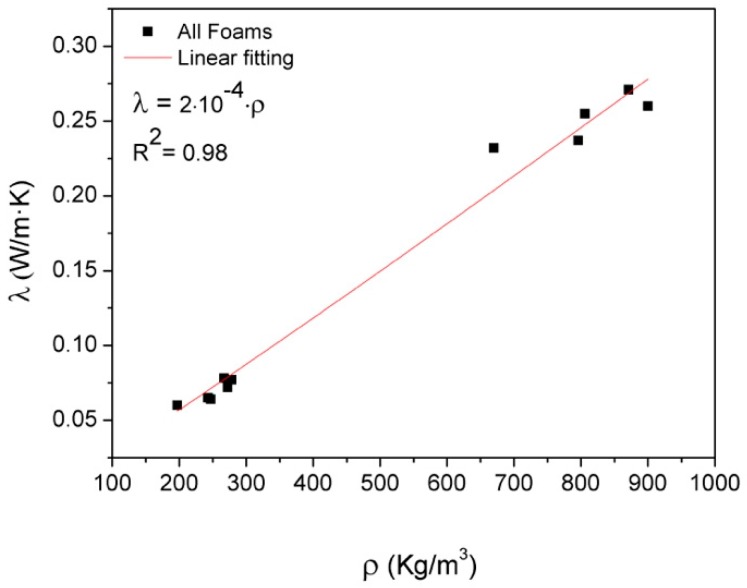
Thermal conductivity as a function of density.

**Figure 7:**
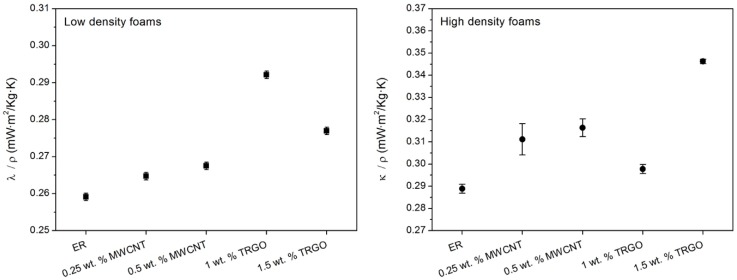
Specific thermal conductivity of (**left**) LD foams and (**right**) HD foams.

The developed nanocomposite foams exhibit very low thermal conductivity values, around 0.07 W/m K for LD and 0.25 W/m K for HD. Figure 6 shows the variation of λ with the foam density, presenting a linear dependence. Hence, in order to eliminate the slight density differences within the low- and high-density sets, the specific value of the conductivity (λ/ρ) is represented in Figure 7. Both nanofillers improve the specific thermal conductivity of the foams with TRGO, showing the largest increase of up to 20% with the addition of 1.5 wt.% of TRGO to HD foams. Such improvements are lower than those previously reported in solid epoxy systems with values above 30% [28,38]. However, the presence of nanoparticles could act as infrared opacifiers [39] affecting the radiative contribution of foams and reducing the thermal conductivity enhancements. This opacifier effect could also explain the downward trend with TRGO loading fraction in LD foams, since these foams are right in the limit where the radiation term starts to contribute to the heat transfer mechanisms [37]. Further studies are currently underway to elucidate such results.

### 3.3. Electrical Conductivity

Among the most sought-after development of nanocomposites filled with carbon-based nanoparticles has been to impart electrical conductivity to the otherwise insulating polymer matrix. A large body of research is available on the subject since it could be exploited in a wide range of applications, from electronics to packaging and aerospace sector. In particular, the development of electrically conductive foams has also been largely studied as they will additionally provide weight reduction to the system. Yang et al. [40,41] and soon after Xu et al. [42] reported PS and PU foams with electrical conductivities of the order of 10^−1^ S/m with 15 wt.% CNF and 560 kg/m^3^, and 10^−5^ S/m with 2 wt.% CNT and 50 kg/m^3^, respectively. Bao and coworkers have more recently reported conductive epoxy foams via a two-step foaming process with electrical resistivity in the order of 10^5^ ohm·cm with 0.5 wt.% CNT and 2 wt.% of EMSs [18], and conductivity in the order of 10^−7^ S/m with 2 wt.% of CNT and of EMSs [20]. Meanwhile, studies carried out with hollow glass microballoons have reached a resistivity of 10^5^ ohm·m with 0.5 vol.% of graphene nanoplatelets and 30 vol.% of hollow glass microballoons [15].

**Figure 8:**
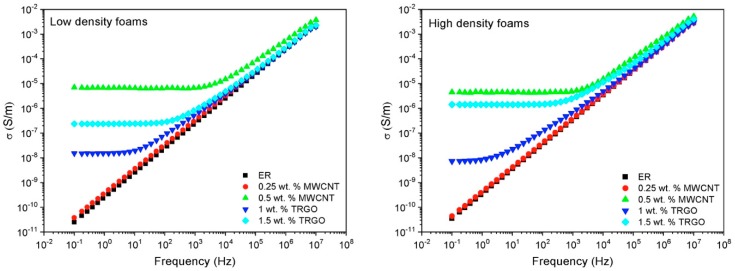
Electrical conductivity of (**left**) LD foams and (**right**) HD foams.

Figure 8 shows the electrical conductivity of the LD and HD foams. The conductivity of the neat epoxy resin shows a linear dependency with the frequency, characteristic of an insulating material. This behavior is modified by the addition of the conductive nanofillers, where the conductivity shows a plateau up to a critical frequency. This behavior is commonly described by the percolation theory [43], which states the existence of a concentration, or percolation threshold, where the filler forms a conductive network. Both concentrations of TRGO (1 wt.% and 1.5 wt.%) and 0.5 wt.% CNT are above the percolation threshold for the two sets of foam densities, where the 0.5 wt.% MWCNT sample shows the highest value of electrical conductivity, close to 10^−5^ S/m. Previous articles by the authors have also observed differences in the percolation threshold and electrical conductivities of CNT and TRGO filled solid epoxy resins, which were ascribed to the geometry of the fillers [26,44], and have later been corroborated by other authors [45].

Finally, both high and low densities present very similar values of electrical conductivities. This result is in agreement with Xu et al. that observed fairly constant electrical conductivities for densities between 200 and 500 kg/m^3^, where the nanofillers formed a 3D percolated network. They then reported a conductivity decrease with density and its disappearing at densities below 30 kg/m^3^ due to a transition from 3D to 2D percolation network and the decrease in CNT content in the cell walls [42]. 

## 4. Conclusions

A one-step foaming process has been optimized using an industrial manufacturing process to produce low and high-density epoxy foams using expandable microspheres. The key assets of the proposed protocol are a very short foaming step followed by the cooling of the mold. The developed foams present enhanced thermal and electrical conductivities, due to the addition of low loading fractions of carbon-based nanoparticles. Here, the addition of 0.5 wt.% of MWCNTs increased the electrical conductivity in five orders of magnitude. While the thermal conductivity was slightly increased, up to 20%, by the nanoparticles in the low and high-density foams. Transport properties showed a different tendency with foam density, where the electrical conductivity was almost constant while the thermal conductivity presented a linear dependency with the density.

These expandable foams could widen the range of applications of syntactic foams as they could be introduced as the core material in the sandwich structures of carbon fiber composites. Additionally, the electrical conductivity of the nanocomposites could represent an asset in those composites, as it would provide a higher electrostatic discharge after a lightning strike. Conductive epoxy foams can also serve as EMI shielding barriers in electronics or in devices inside the aircraft, due to their lightweight.

## Figures and Tables

**Table 1:** Dispersing protocol.

Protocol	Gap 1 (µm)	Gap 2 (µm)	Speed (rpm)	Time (min)
Step 1	120	40	100	10
Step 2	60	20	80	10
Step 3	15	5	80	10

**Table 2:** Cell size and densities of the epoxy foams.

Sample	Cell Size (µm)	*ρ* (kg/m^3^)
ER-LD	130 ± 29	247 ± 3
0.25 wt.% MWCNT-LD	118 ± 41	272 ± 2
0.5 wt.% MWCNT-LD	129 ± 36	243 ± 1
1 wt.% TRGO-LD	126 ± 35	267 ± 3
1.5 wt.% TRGO-LD	125 ± 31	278 ± 1
ER-HD	58 ± 27	707 ± 1
0.25 wt.% MWCNT-HD	53 ± 17	871 ± 3
0.5 wt.% MWCNT-HD	55 ± 28	806 ± 2
1 wt.% TRGO-HD	56 ± 17	796 ± 1
1.5 wt.% TRGO-HD	57 ± 26	670 ± 1
